# How Much Burnout and Coping Influence Quality of Life among Young Oncology Providers in Romania during the COVID-19 Pandemic

**DOI:** 10.3390/ijerph19095508

**Published:** 2022-05-01

**Authors:** Cristian-Virgil Lungulescu, Adina Turcu-Stiolica, Cristina Lungulescu, Elena-Adriana Dumitrescu, Razvan-Aurelian Turcu-Stiolica, Vlad-Mihai Croitoru, Irina-Mihaela Cazacu, Adelina-Silvana Gheorghe, Dana-Lucia Stanculeanu, Daniel Sur

**Affiliations:** 1 Oncology Department, University of Medicine and Pharmacy of Craiova, 2-4 Petru Rares Str., 200349 Craiova, Romania; cristilungulescu@yahoo.com; 2 Pharmacoeconomics Department, University of Medicine and Pharmacy of Craiova, 2-4 Petru Rares Str., 200349 Craiova, Romania; 3 Doctoral School, University of Medicine and Pharmacy of Craiova, 2-4 Petru Rares Str., 200349 Craiova, Romania; cristina.lungulescu@yahoo.com; 4 Institute of Oncology, Prof. Dr. Alexandru Trestioreanu, Șoseaua Fundeni, 022328 Bucharest, Romania; elena-adriana.dumitrescu@drd.umfcd.ro (E.-A.D.); adelina-silvana.gheorghe@drd.umfcd.ro (A.-S.G.); dana.stanculeanu@umfcd.ro (D.-L.S.); 5 Psihology Department, “Lucian Blaga” University of Sibiu, Bld. Victoriei 10, 550024 Sibiu, Romania; razvan.turcu@gmail.com; 6 Department of Oncology, Fundeni Clinical Institute, 258 Fundeni Str., 022238 Bucharest, Romania; vlad.m.croitoru@gmail.com (V.-M.C.); irina.cazacu89@gmail.com (I.-M.C.); 7 11th Department of Medical Oncology, University of Medicine and Pharmacy Iuliu Hatieganu, 400012 Cluj-Napoca, Romania; dr.geni@yahoo.co.uk

**Keywords:** burnout, coping, quality of life, young oncologists, COVID-19

## Abstract

This study aims to investigate the correlations between burnout, coping strategies, and quality of life among young oncology healthcare workers in Romania during the COVID-19 pandemic. We collected the data using an online questionnaire consisting of sociodemographic questions, the Maslach Burnout Inventory, the COPE questionnaire, and the 15D instrument. A total of 122 healthcare providers responded to our survey. We evaluated the differences in the scores among the three groups of healthcare workers in oncology under 40 years old: medical oncologists (n = 87), radiation oncologists (n = 11), and oncology nurses (n = 24). Finally, we conducted a correlation analysis between the dimensions of burnout, coping, and quality of life. Overall, the medical oncologists exhibited much higher burnout levels than nurses in the COVID-19 pandemic outbreak, having statistically significant higher levels of emotional exhaustion, depersonalization, and lack of personal achievement. Some factors were inversely associated with burnout: active approach, planning, positive interpretation and growth, and acceptance. Our findings illustrated a very good level of health-related quality of life (average = 0.93, SD = 0.06), and no statistically significant differences were found in the quality of life between the three groups. This study was the first to identify the profile of young oncology providers in Romania. Our findings may be relevant in creating preventive strategies for burnout and increasing the quality of life in Romanian young oncology providers in future crises.

## 1. Introduction

In recent years, it has become clear how much the emotional well-being of health professionals affects the quality of patient care. Cancer physicians in particular are known to be at particular risk of developing burnout syndrome, largely due to direct contact with critically ill patients and their families and an ever-changing medical landscape [1,2].

Burnout was first described in the 1970s as a stress-induced, occupational-related syndrome characterized by emotional exhaustion, depersonalization, and a lack of personal accomplishment [3,4]. Burnout is common in oncology, with a prevalence of 25–35% among medical oncologists, 28% among radiation oncologists, and 28–36% among surgical oncologists [5].

Although physicians with the highest amount of burnout are more likely to develop major depression, workers often have one without the other, as highlighted by Maslach et al. when first describing the Maslach Burnout Inventory, and again more recently [6]. Regardless of the diagnostic category, psychological and behavioral changes in oncology must be assessed by qualified mental health professionals in a nonjudgmental and comprehensive manner. To date, there is no formal, tailored clinical diagnosis to identify burnout symptoms in individual oncologists. Nevertheless, self-reported screening tools such as the Maslach Burnout Inventory have been used in research and organizations for oncology clinicians [7].

Young oncology providers, defined as medical oncologists, radiation oncologists, and nurses under 40 years of age, are a special risk group, due to their heavy workload, academic pressure, increasing administrative tasks, constant life-and-death decisions, administration of potentially toxic therapies, and facing legal problems in a time of reduced resources [8,9]. Reports indicate that burnout was associated with being younger, reduced psychological well-being, difficulties outside of work, workplace demands, and workplace stress [10,11]. Previous studies have not specifically examined burnout rates among younger oncologists. This group of oncologists is key to the future oncology workforce to provide patient care, advance research, and train the next generation of oncologists.

Therefore, it is important to establish the extent of burnout in this group and identify interventions that support young oncology providers coping with mental health problems. The good news is that more and more attention is being paid to the underlying problem and to finding creative solutions. The way forward is still open but will most likely involve solving the problems at the root, as well as smart dissemination and implementation at the individual and systemic levels.

The aim of the Romanian Young Oncology Committee survey was to investigate the burnout prevalence, quality of life, and coping methods amongst Romanian young oncology providers during the COVID-19 pandemic.

## 2. Materials and Methods

### 2.1. Study Design

All oncology doctors and nurses registered in Romania with the age of under 40 were considered eligible for our study to answer the questionnaire. The protocol was approved by the Ethics Committee of the University of Medicine and Pharmacy of Craiova (No. 199/24.11.2021) according to the requirements of the Helsinki Declaration. All the participants provided electronic informed consent responding to the first question of the survey.

The study was performed between 1 November and 15 December 2021. After consenting, the participants responded to the online survey, including sociodemographic questions (gender, age, marital status), three questions regarding occupation (specialty, experience, primary place of work), The Maslach Burnout Inventory, and the COPE and 15D questionnaires. The online survey was developed using Google Forms and it was self-administered, anonymous, and confidential, with all questions being obligatory (no missing data). As dissemination channels, we used the database from the Romanian National Society of Medical Oncology and different communication channels for Young Oncologists on social media. The medical staff in Romania in medical oncology are mostly represented by personnel over 40, and only now has there been a generational change that has allowed us to distribute the questionnaire. The data collection of this current study was conducted by the Young Oncologists Division of the Romanian National Society of Medical Oncology on a representative sample of the Association’s young active members (122 of 180 members), represented mainly by medical oncologists (87 of them). The other two categories of the cohort (nurses and radiation oncologists) are less affiliated with the Young Oncologists Division Association, explaining the low participation of these two subgroups of oncology providers. The percentage of oncology doctors and nurses that actually filled out the survey was 68%.

### 2.2. Instruments

The Maslach Burnout Inventory questionnaire was designed to merge three psychological parameters (emotional exhaustion (EE), depersonalization (DP), lack of personal achievement (PA)) into one final score. According to the Burnout Score, the respondent has low (25–50), medium (51–75), or high (76–125) burnout. The three dimensions could also be interpreted for their values and the scores permit the interpretation of the burnout: emotional exhaustion (low 9–18, medium 19–27, high 28–45), depersonalization (low 6–12, medium 13–18, high 19–30), and lack of personal achievement (low 10–20, medium 21–30, high 31–50). We used the translated and validated Romanian questionnaire [12].

The COPE Questionnaire assesses 14 coping strategies [13] which identify 4 factors: coping focalized on the problem (active approach, planning, deletion of concurrent activities), coping focalized on emotions (positive interpretation, restraint, acceptance, religious approach), coping focalized on the search for social support (use of social-instrumental support, use of social-emotional support, expressing the emotions), and avoidance coping for the associated emotions (denial, mental deactivation, behavioral deactivation). One coping scale evaluates substance consumption (anxiolytic medication or alcohol) to eliminate the confrontation with threatening situations, in our case the COVID-19 pandemic. The questionnaire has 53 items, and the answers are measured on a Likert scale from 1 to 4. The Romanian version of the COPE Questionnaire demonstrated high internal consistency with a Cronbach’s alpha coefficient of 0.74.

The quality of life was evaluated using the Romanian version of the 15D instrument [14,15] through 15 dimensions (mobility, vision, hearing, breathing, sleeping, eating, speech, excretion, usual activities, mental function, discomfort and symptoms, depression, distress, vitality, and sexual activity). The quality of every dimension was calculated from 0 to 1, where 1 represents full health regarding the dimension. The 15D score has a value from 0 to 1, where 1 represents full health and 0 is dead.

### 2.3. Statistical Analysis

The characteristics of the sample were assessed using descriptive statistics. The continuous variables were expressed using mean and standard deviation (SD), or median and interquartile range (IQR). The Mann–Whitney test was used to compare the average burnout, coping strategies, or quality of life for different professional categories (Group 1 = medical oncology, Group 2 = radiation oncology, Group 3 = nurses). Where the differences were statistically significant, the Bonferroni correction was used. The discrete and nominal variables were expressed using frequencies and percentages. The χ2 test or Fisher’s exact test were used to examine differences between the groups for discrete variables. Data were analyzed using GraphPad Prism 9.3.1 (GraphPad Software, San Diego, CA, USA). The power analysis for our study was performed using G*Power 3.1.9.7, at a 95% confidence level and power factor of 80% for each of the groups. All the tests where the *p*-value was less than 0.05 were considered statistically significant.

## 3. Results

### 3.1. Sample Characteristics of Participants

A total of 122 health professionals participated in the study, with demographic information as shown in Table 1. Among the respondents, 87 (71.31%) were medical oncologists (Group 1), 11 (9.02%) were radiation oncologists (Group 2), and 24 (19.67%) were nurses (Group 3). At the time of the study, most of the sample worked in public hospitals (92, 75.41%). The mean age was 30.09 years (SD = 3.81) and most of them were females (95, 77.87%).

### 3.2. Results of Comparisons between the Groups of Participants

The mean burnout score in the sample was 54.02 (SD = 15.94, range = 28–98), with the mean of the psychological parameters for EE: 22.16 (SD = 7.4, range = 9–40), DP: 11.14 (SD = 4.34, range = 6–22), and reduction in PA: 20.72 (SD = 6.35, range = 10–37).

According to the burden scores, as shown in Table 2, we found a high level of EE in the medical oncology group in 22.99%, in the radiation oncology group in 27.27%, and in the nurses’ group in 12.50%. Additionally, a high level of DP was found in 9.20% of medical oncologists, 18.18% of radiation oncologists, and 0% of nurses. A high level of PA was found in 8.05% of medical oncologists, 9.09% of radiation oncologists, and 4.17% of nurses.

**Figure 1:**
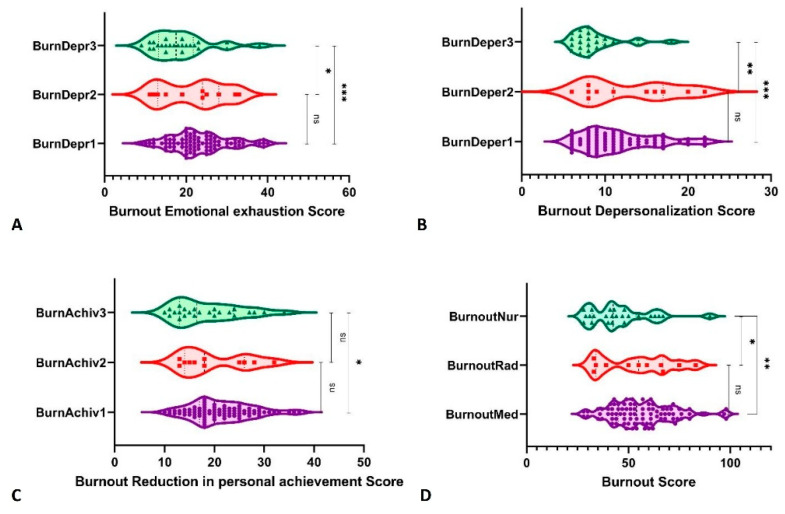
Burnout comparisons between the groups of participants (1 = medical oncology, 2 = radiation oncology, 3 = nurses). *, *p*-value < 0.05; **, *p*-value < 0.01; ***, *p*-value < 0.001; ns, not significant.

We found statistically significant differences among the professional categories (Table 3) for the total score of burnout between medical oncologists and nurses, with higher burnout in doctors than in nurses (56.3 ± 15.36 vs. 45.75 ± 15.09, *p*-value = 0.0014), as in Figure 1. The same differences were maintained for the three dimensions of burnout: more EE for medical oncologists than nurses (23.28 ± 7.20 vs. 18.42 ± 6.88, *p*-value = 0.0012), more DP (11.59 ± 4.32 vs. 8.79 ± 2.93, *p*-value = 0.001), and more PA (21.44 ± 6.15 vs. 18.54 ± 6.63, *p*-value = 0.034).

There were no statistically significant differences in burnout between the two types of specialty of doctors or between radiation oncologists and nurses. Only the dimension of depersonalization was significantly higher for radiation oncologists than nurses (12.73 ± 5.5 vs. 8.79 ± 2.93, *p*-value = 0.03). 

**Figure 2:**
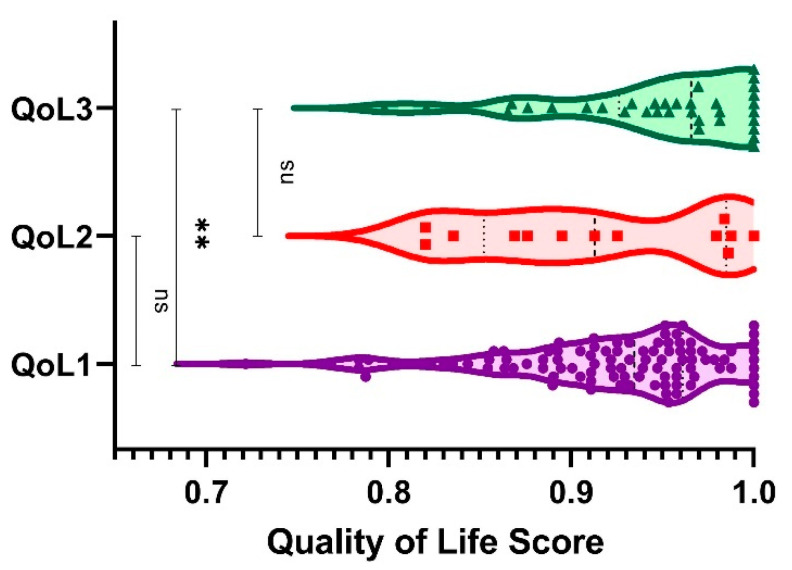
Quality of life comparison between the groups of participants (1 = medical oncology, 2 = radiation oncology, 3 = nurses). **, *p*-value < 0.01; ns, not significant.

The mean total QoL was 0.93 (SD = 0.06, range = 0.72–1.00) according to the mean directions for sleeping: 0.81 (SD = 0.2, range = 0.30–1), speech: 0.88 (SD = 0.16, range = 0.43–1), mental: 0.89 (SD = 0.17, range = 0.38–1), depression: 0.82 (SD = 0.18, range = 0.31–1), distress: 0.71 (SD = 0.21, range = 0.13–1), vitality: 0.77 (SD = 0.19, range = 0.13–1), and sexual activity: 0.83 (SD = 0.23, range = 0.13–1). The QoL score comparisons between the groups of participants are shown in Figure 2.

Among the professional categories, no statistically significant differences emerged for the coping subscales. Only religious approach was more often used as a coping strategy by nurses than by medical oncologists (11.46 ± 3.83 vs. 9.48 ± 4.21, *p*-value = 0.047).

Table 3 also shows aspects related to quality of life and the dimensions related to it. The quality of life was found to be higher for nurses than for doctors, but not statistically significant. Nevertheless, speech (0.95 ± 0.11 vs. 0.86 ± 0.17, *p*-value = 0.023), mental health (0.97 ± 0.1 vs. 0.87 ± 0.18, *p*-value = 0.03), and distress (0.78 ± 0.24 vs. 0.69 ± 0.19, *p*-value = 0.029) had better scores for nurses than for medical oncologists.

**Figure 3:**
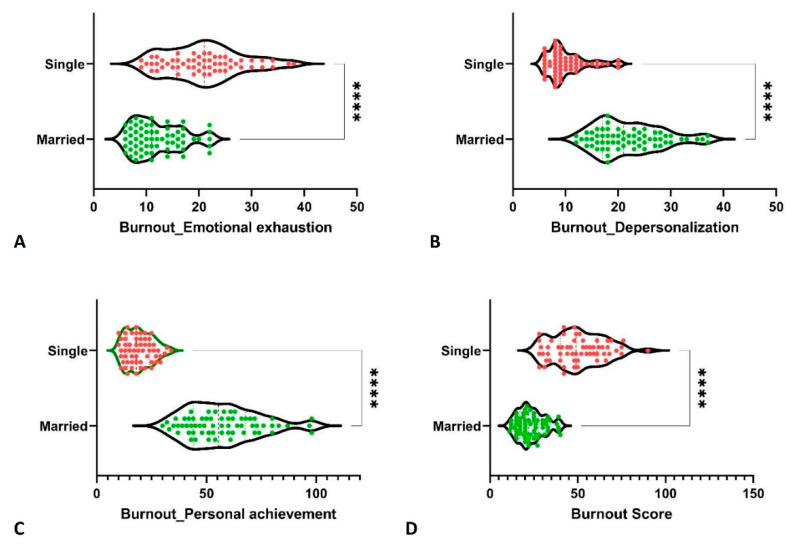
Burnout comparison between married and single participants. ****, *p*-value < 0.0001.

According to marital status, the single participants had higher burnout than married ones (50.31 ± 14.56 vs. 23.33 ± 7.49, *p*-value < 0.0001), a difference that was supported by all three characteristics: higher EE for single participants (20.86 ± 7.15 vs. 12.05 ± 4.68, *p*-value < 0.0001), higher DP for married participants (22.00 ± 6.35 vs. 10.14 ± 3.73, *p*-value < 0.0001), and higher PA for married participants (57.38 ± 16.50 vs. 19.31 ± 6.1, *p*-value < 0.0001), as shown in Figure 3.

No differences of burnout were found among participants according to gender, where the same values were observed for men and women for burnout score (51 ± 16.17 vs. 54.87 ± 15.86, *p*-value = 0.2180) and its characteristics: EE (20.37 ± 7.32 vs. 22.66 ± 7.39, *p*-value = 0.1758), DP (11.67 ± 4.79 vs. 10.99 ± 4.22, *p*-value = 0.5788), and PA (18.96 ± 5.59 vs. 21.22 ± 6.52, *p*-value = 0.0774).

### 3.3. Results of Correlations between Burnout, Coping Strategies, and QoL

Investigating correlations between burnout and coping, our results demonstrated positive correlations between burnout and denial (ρ = 0.335, *p*-value < 0.0001), expressing (ρ = 0.354, *p*-value < 0.0001), passive mental (ρ = 0.353, *p*-value < 0.0001), and passive behavior (ρ = 0.640, *p*-value < 0.0001), as in Table 4. The personal burnout had inverse correlations with active coping (ρ = −0.505, *p*-value < 0.0001), planning (ρ = −0.456, *p*-value < 0.0001), reframing (ρ = −0.468, *p*-value < 0.0001), and acceptance (ρ = −0.202, *p*-value = 0.025) subscales, respectively. Moreover, the burnout had inverse correlations with QoL dimensions such as sleeping (ρ = −0.357, *p*-value < 0.0001), speech (ρ = −0.380, *p*-value < 0.0001), mental (ρ = −0.571, *p*-value < 0.0001), depression (ρ = −0.652, *p*-value < 0.0001), distress (ρ = −0.550, *p*-value < 0.0001), vitality (ρ = −0.593, *p*-value < 0.0001), and sex (ρ = −0.516, *p*-value < 0.0001) subscales, as well as QoL score (ρ = −0.725, *p*-value < 0.0001), respectively.

**Figure 4:**
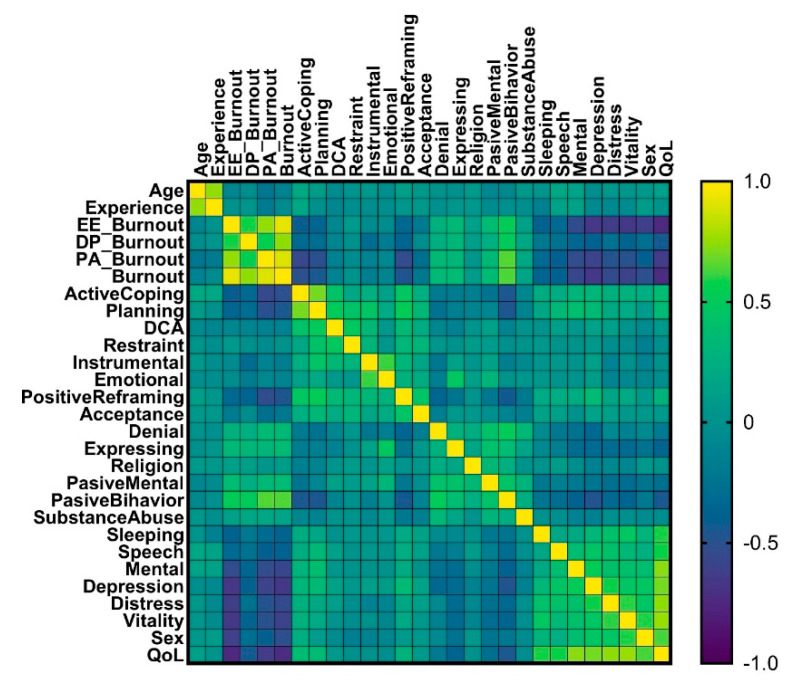
Heatmap matrix.

All the correlations between the measured indicators are presented in Figure 4.

## 4. Discussion

This research aimed to assess the burnout, the coping strategies, and the quality of life among young Romanian healthcare workers (physicians and nurses) working in oncology departments in hospitals during the COVID-19 pandemic. According to Romanian government records, the total number of patients hospitalized with COVID-19 in specialized health institutions at the beginning of the research, on 1 November 2021, was 20,561. A total of 1876 of the patients were admitted to intensive care units [16]. The time period covered by this study corresponds to the fourth wave of the pandemic in Romania. The performance and well-being of physicians and nurses are negatively impacted by psychological distress and burnout, which have long been recognized as problems in the field. It is also important to acquire data from a new geographic area that has not been previously investigated.

The mean burnout score for all healthcare providers surveyed in the current study was 54.02, indicating a moderate level of burnout, according to the Maslach Burnout Inventory. These findings are consistent with earlier research that has found moderate burnout among physicians and nurses during the COVID-19 pandemic. AlJhani et al. found significant personal and work-related burnout (mean 58.9 and 56.9, respectively) among doctors and nurses in Saudi Arabia, according to descriptive statistics of the Copenhagen Burnout Inventory (CBI) dimensions and Brief COPE [17]. Similarly, after utilizing CBI to examine 933 Malaysian healthcare workers, personal and work-related burnout was 53.8 and 39.1, respectively [18]. More than half of the HCWs in Portugal, according to another study, have faced significant levels of burnout, both personally and professionally [19]. Burnout criteria were met by over 70% of HCWs in Singapore during the pandemic [20], and over 50% of a total of 2707 HCWs from 60 different countries [21]. Significant burnout has been a common occurrence among HCWs, although other studies have found moderate to minor burnout [22,23].

In the present study, doctors were shown to be more severely impacted by burnout than nurses (56.3 ± 15.36 vs. 45.75 ± 15.09, *p*-value = 0.0014). After assessing the three components of burnout, the findings remained consistent for medical oncologists compared to nurses: more emotional tiredness (*p*-value = 0.0012), greater depersonalization (*p*-value = 0.001), and reduced personal accomplishment (*p*-value = 0.034). 

Concerning the quality of life, our study shows that nurses had a higher quality of life than physicians, although the difference was not statistically significant. Nevertheless, speech (*p*-value = 0.023), mental health (*p*-value = 0.03), and distress (*p*-value = 0.029), assessed using the Romanian version of the 15D instrument, had better scores for nurses than for medical oncologists. The quality of life of young Romanian physicians in the COVID-19 outbreak was assessed among gastroenterologists [24]. They obtained similar results, with a mean value of QoL (median, IQR) of 0.97 (0.06) for young gastroenterologists.

In terms of coping, our findings revealed positive relationships between burnout and denial (ρ = 0.335, *p*-value < 0.0001), expressing (ρ = 0.354, *p*-value < 0.0001), passive mental (ρ = 0.353, *p*-value < 0.0001), and passive behavior (ρ = 0.640, *p*-value < 0.0001). Personal burnout exhibited inverse associations with the subscales assessing active coping (ρ = −0.505, *p*-value < 0.0001), planning (ρ = −0.456, *p*-value < 0.0001), reframing (ρ = −0.468, *p*-value < 0.0001), and acceptance (ρ = −0.202, *p*-value = 0.025). For the coping subscales, there were no statistically significant differences across professional groups. A statistically significant difference existed in the use of religious methods as coping mechanisms between nurses and medical oncologists (*p*-value = 0.047), with nurses being more likely to use religious approaches. Spirituality had the greatest mean score for adaptive coping among healthcare workers in Saudi Arabia. Furthermore, there was a negative correlation between work-related burnout and religion [17].

Our findings corroborate previously published research [17,25,26]. Avoidance coping appeared as the greatest indicator of the quality of life and well-being, making it an excellent target for treatments [25,27,28]. Similarly, others have discovered an association between emotional and avoidance coping and stress, anxiety, and depression [29].

Interestingly, several studies utilizing the Maslach Burnout Inventory indicated that burnout was more common among nurses than among physicians [30,31]. Previous research involving healthcare workers, which included physicians, nurses, and administrative and support personnel, discovered that higher education levels were associated with higher degrees of burnout [20]. One probable reason is that it is related to seniority in healthcare and therefore increased responsibility.

Furthermore, evidence suggests that the pandemic has had an uneven impact on gender and that the higher weight of family duties placed on female doctors may be increasing professional pressures [26,32,33]. A previous study where women made up the majority of participants (83.6%) also suggests that female sex is related to a greater degree of personal burnout [19], data consistent with other reports [34,35]. Additionally, nurses are more prone to experience burnout than other caregivers due to their gender (primarily females) and the fact that they are the largest caregiver group providing front-line assistance, both elements being risk factors leading to burnout [36,37].

Conversely, in a study involving 200 physicians in Turkey (59% women and 41% men) using the Maslach Burnout Inventory, researchers found that the level of burnout did not vary dramatically between men and women [26]. This conclusion is in line with prior research, which revealed that the prevalence of burnout was comparable between genders. Similarly, our research found no difference in terms of burnout between men and women [38,39].

Between medical oncologists and radiation oncologists, there were no statistically significant variations in burnout levels found in our study. Surprisingly, reports from Melbourne, Australia, where the pandemic was especially devastating, show that radiation oncologists overwhelmingly endorsed changes to practice in response to the pandemic [40]. When proper information technology and childcare help are accessible (the vast majority of radiation oncologists were female), the opportunity to work from home has been regarded as a good experience that is related to reduced burnout [41].

Numerous data from the literature indicate that younger healthcare professionals are more susceptible to burnout than their more experienced counterparts [26]. Several studies determined that individuals with less years of professional experience in the medical setting were more vulnerable to suffering burnout than individuals with a longer tenure in the profession [17,31]. Specifically, burnout was more frequent among HCWs aged 22–34 years than those over 35 years old [17]. Similarly, it was reported that the risk of burnout is greater for healthcare workers during their first two years of training [42].

Healthcare workers under the age of 40 remain at a greater risk of burnout and stress, according to the ESMO Resilience Task Force survey series [26], which assessed participants with an average of 15 years of experience working in oncology (with medical oncology accounting for 72% of the total) from 104 countries. Similarly, elder respondents (50–65 years) reported higher well-being than younger respondents (16–29 years). A longer career may mean a greater capacity to cope with work-related strain [25].

Intriguingly, higher EE and DP levels were associated with younger participants (*p* = 0.05 for both), whereas lower PA scores were strongly linked to lack of experience (*p* = 0.003), according to research conducted on various specialties of Egyptian physicians [34]. Moreover, in terms of Burnout Syndrome (BOS) domains, a younger age was associated with the development of BOS in general and the DP component in particular [34]. These findings are also consistent with pre-pandemic research indicating that burnout levels in doctors tend to decrease with seniority [43,44].

Grouping based on marital status, we found higher burnout among single participants, having a direct positive correlation with EE and an inverse correlation with DP and PA. The syndrome of emotional exhaustion was more intense in single participants during the COVID-19 pandemic, in the presence of rumination. At the same time, the presence of a life partner involved an additional stressor, with an impact on the feelings of failure and helplessness, with higher DP and PA. One study involving 83.6% female healthcare workers hypothesized that higher burnout among married participants might be connected to the dual role that married women play [19]. With contradicting reports, the relationship between burnout syndrome and marital status is not well-established thus far [18].

Research shows that oncology HCWs express increasing concerns over declining professional training opportunities (43%), foreign fellowships (76%), and overall job security (37%). A quarter of responders are thinking about altering their career prospects, while 38% ponder abandoning the profession [26]. These prospects are alarming when taking into account that young physicians are particularly susceptible to burnout syndrome. Additionally, it is perceived that the number of clinical trials and research initiatives is declining [26].

However, it is worth noting that international initiatives such as the introduction of virtual oncology fellowships and hosting specialized webinars have received positive feedback [45,46]. It is believed that these approaches may reduce financial expenses and increase the participation of WCPs who struggle with other responsibilities.

As a result of increased workload, job strain, and time constraints, and a lack of institutional assistance, burnout is more prevalent among healthcare workers during the COVID-19 pandemic [22]. In addition to greater training opportunities, organizations should also provide assistance for family members, personal protective equipment, and mental health programs to reduce immediate and potential burnout among HCPs. Furthermore, resilience training, such as proactive coping, planning, positive reframing, emotional, and instrumental support, may provide HCWs with the necessary assistance during the COVID-19 pandemic [47], and some psychological support models are already being proposed for future crises [48].

This research has certain limitations. Firstly, the findings were drawn from a small sample size, which might have a detrimental influence on generalization. The power test was performed, and assuming an alpha level of 0.05, the participants from the three groups yielded a power between 78% and 91% for the different analyses. Secondly, the cross-sectional approach precludes us from establishing a causal relationship between the variables under research and from drawing conclusions about the long-term consequences of coping. Furthermore, given that most of the participants were female (77.87%), establishing generalizations about male HCWs is challenging. Even though this study centers on the relationship between distress and coping, other variables, such as different healthcare systems and resources, may impact those factors. Using an online survey may have restricted access to individuals who are less inclined to use the internet. However, online surveys continue to be the most effective and secure method of reaching a larger population given the pandemic’s restrictions. The research was conducted over a one-and-a-half-month period and is only relevant to a specific pandemic timeframe, coinciding with the gradual relaxation of lockdown measures in the affected areas. It is possible that future studies may examine the degrees of burnout experienced by clinicians when the pandemic is deemed under control and provide a basis for comparison.

Given the unexpected and continuing nature of the pandemic, it might be recommended to monitor HCWs’ mental health in order to enhance their quality of life by promoting awareness. The employment of adaptive coping strategies also indicates psychological well-being, which further results in better and safer practices [49].

## 5. Conclusions

In conclusion, young oncology physicians suffered medium–high burnout in a high percentage, which negatively correlated with their quality of life, during the COVID-19 pandemic in Romania. These findings, together with the coping strategies HCWs preferred, may be relevant in creating preventive strategies for burnout for young oncology providers in Romania. 

## Figures and Tables

**Table 1:** Participants’ demographic characteristics (n = 122).

Characteristics	Mean (±SD), Median (IQR), or n (%)
Age (years)	30.09 (±3.81), 29.5 (27.0–33.0)
Gender	
Female	95 (77.9%)
Male	27 (22.1%)
Marital status	
Married	54 (44.3%)
Single	64 (52.5%)
Divorced	4 (3.3%)
Specialty	
Medical oncology	87 (71.3%)
Radiation oncology	11 (9.0%)
Nurses	24 (19.7%)
Experience (years)	4.34 (±3.58), 3 (2–5)
Primary place of work	
General hospital	92 (75.41%)
Private clinic	30 (24.59%)

**Table 2:** Distribution of the sample for the burnout level.

n (%)	Medical Oncology	Radiation Oncology	Nurses
	(n = 87)	(n = 11)	(n = 24)
	Group 1	Group 2	Group 3
Emotional exhaustion			
Low (≤18)	21 (24.14%)	4 (36.36%)	13 (54.17%)
Medium (19–27)	46 (52.87%)	4 (36.36%)	8 (33.33%)
High (≥28)	20 (22.99%)	3 (27.27%)	3 (12.50%)
Depersonalization			
Low (≤12)	59 (67.82%)	6 (54.55%)	21 (87.50%)
Medium (13–18)	20 (22.99%)	3 (27.27%)	3 (12.50%)
High (≥19)	8 (9.20%)	2 (18.18%)	0
Lack of personal achievement			
Low (≤20)	44 (50.57%)	7 (63.64%)	16 (66.67%)
Medium (21–30)	36 (41.38%)	3 (27.27%)	7 (29.17%)
High (≥31)	7 (8.05%)	1 (9.09%)	1 (4.17%)
Burnout score			
Low (≤50)	33 (37.93%)	5 (45.45%)	18 (75.00%)
Medium (51–75)	45 (51.72%)	5 (45.45%)	5 (20.83%)
High (≥76)	9 (10.34%)	1 (9.09%)	1 (4.17%)

**Table 3:** Descriptive statistics of Burnout, COPE, and QoL, and comparisons between the groups.

Mean ± SD, Median (IQR)	Medical Oncology (n = 87) Group 1	Radiation Oncology (n = 11) Group 2	Nurses (n = 24) Group 3	*p*-Value Group 1 vs. Group 2	*p*-Value Group 1 vs. Group 3	*p*-Value Group 2 vs. Group 3
Age	29.75 ± 2.99 29 (27–32)	31.09 ± 3.67 30 (28–33)	30.88 ± 5.98 29 (26.3–37.8)	0.223	0.658	0.556
Experience	3.68 ± 2.47 3 (2–5)	4.45 ± 1.64 5 (3–6)	6.71 ± 5.99 4.5 (2–11.5)	0.119	0.062	0.900
Burnout Questionnaire						
Emotional exhaustion	23.28 ± 7.20 22 (19–27)	21.45 ± 7.94 24 (13–28)	18.42 ± 6.88 17.5 (13.3–21.8)	0.517	0.0012 **	0.255
Depersonalization	11.59 ± 4.32 11 (8–14)	12.73 ± 5.5 11 (8–17)	8.79 ± 2.93 8 (7–9.75)	0.694	0.001 ***	0.03 *
Lack of personal achievement	21.44 ± 6.15 20 (17–25)	19.82 ± 6.72 18 (14–26)	18.54 ± 6.63 16.5 (13–23.75)	0.335	0.034 *	0.533
Burnout Score	56.3 ± 15.36 54 (45–66)	54 ± 17.73 55 (34–67)	45.75 ± 15.09 42.5 (32.3–53.8)	0.687	0.0014 **	0.149
COPE Questionnaire						
Adaptive coping subscales						
Active approach (4/16)	13.02 ± 1.98 13 (12–15)	13.36 ± 1.86 13 (12–15)	13.08 ± 2.10 13 (11.25–15)	0.580	0.862	0.759
Planning (4/16)	13.92 ± 2.33 14 (12–16)	14.36 ± 1.96 15 (13–16)	13.96 ± 2.4 15 (12–16)	0.696	0.953	0.839
Deletion of concurrent activities	11.55 ± 1.87 12 (10–13)	11.73 ± 1.49 12 (10–13)	11.25 ± 2.58 11 (10–13)	0.820	0.682	0.653
Restraint	10.24 ± 2.41 10 (9–12)	11.45 ± 2.02 11 (9–13)	11 ± 2.45 11 (9–13)	0.140	0.254	0.591
Use of social-instrumental support	13.02 ± 2.7 14 (11–15)	13.18 ± 2.72 12 (11–16)	12.75 ± 2.09 12 (11–14.75)	0.968	0.351	0.665
Use of social-emotional support	11.85 ± 3.41 12 (10–15)	12.91 ± 2.26 13 (12–15)	11.13 ± 2.97 11.5 (9–14)	0.435	0.199	0.095
Positive interpretation and growth	13.75 ± 2.21 14 (12–16)	14.91 ± 1.76 16 (15–16)	13.83 ± 2.14 14 (12–16)	0.074	0.889	0.174
Acceptance	12.77 ± 2.46 13 (11–15)	13.55 ± 2.21 13 (12–16)	12.25 ± 2.52 12.5 (10–14.75)	0.420	0.283	0.155
Religious approach	9.48 ± 4.21 10 (5–13)	10.64 ± 4.52 12 (5–15)	11.46 ± 3.83 12 (8.75–14.75)	0.356	0.047 *	0.707
Maladaptive coping subscales						
Denial	6.16 ± 2.23 6 (4–7)	6.27 ± 2.10 5 (5–8)	7.08 ± 2.8 6.5 (5–9)	0.748	0.147	0.493
Expressing the emotions	9.25 ± 2.96 9 (7–11)	8 ± 2.24 7 (7–10)	8.92 ± 3.28 8.5 (6–12)	0.181	0.572	0.567
Mental deactivation	8.95 ± 2.62 9 (8–11)	8.00 ± 2.68 8 (5–11)	8.50 ± 2.8 8 (6.25–11)	0.285	0.376	0.667
Behavioral deactivation (4/16)	6.44 ± 2.49 6 (5–7)	6.64 ± 2.11 6 (5–8)	6.42 ± 2.48 5.5 (4.25–8)	0.522	0.896	0.577
Substance abuse (1/4)	1.31 ± 0.7 1 (1–1)	1.18 ± 0.6 1 (1–1)	1.46 ± 0.88 1 (1–1)	0.438	0.497	0.296
Quality of Life questionnaire						
Sleeping	0.81 ± 0.19 0.76 (0.76–1)	0.86 ± 0.15 1 (0.7–1)	0.79 ± 0.23 0.76 (0.51–1)	0.453	0.591	0.367
Speech	0.86 ± 0.17 1 (0.7–1)	0.84 ± 0.15 0.7 (0.7–1)	0.95 ± 0.11 1 (1–1)	0.902	0.023 *	0.075
Mental function	0.87 ± 0.18 1 (0.6–1)	0.84 ± 0.19 1 (0.64–1)	0.97 ± 0.1 1 (1–1)	0.984	0.030 *	0.106
Depression	0.82 ± 0.17 0.76 (0.76–1)	0.85 ± 0.16 0.76 (0.76–1)	0.86 ± 0.19 1 (0.7–1)	0.222	0.451	0.626
Distress	0.69 ± 0.19 0.7 (0.48–0.7)	0.71 ± 0.22 0.72 (0.48–1)	0.78 ± 0.24 0.72 (0.66–1)	0.317	0.029 *	0.985
Vitality	0.76 ± 0.19 0.77 (0.7–0.8)	0.76 ± 0.19 0.77 (0.52–1)	0.82 ± 0.19 0.77 (0.77–1)	0.418	0.199	0.923
Sexual activity	0.82 ± 0.23 1 (0.7–1)	0.78 ± 0.2 0.7 (0.7–1)	0.89 ± 0.18 1 (0.71–1)	0.608	0.277	0.221
QoL score	0.92 ± 0.06 0.93 (0.9–0.96)	0.91 ± 0.07 0.9 (0.8–0.98)	0.95 ± 0.05 0.9 (0.9–1)	0.562	0.065	0.486

The dimensions measured with the 15D instrument that had 1 for all participants are not shown in the table (mobility, vision, hearing, breathing, eating, excretion, usual activities, discomfort and symptoms). *, *p*-value < 0.05; **, *p*-value < 0.01; ***, *p*-value < 0.001.

**Table 4:** Correlations between the burnout dimensions and COPE/QoL domains.

COPE and QoL Domains	QoL	Burnout			
		Emotional Exhaustion	Depersonalization	Lack of Personal Accomplishment	Total Score
Active approach	0.360 ****	−0.415 ****	−0.322 ****	−0.557 ****	−0.505 ****
Planning	0.354 ****	−0.346 ****	−0.296 ***	−0.513 ****	−0.456 ****
Deletion of concurrent activities	0.082	−0.080	−0.099	−0.131	−0.125
Restraint	−0.010	0.067	−0.010	−0.049	0.004
Use of social-instrumental support	−0.002	−0.052	−0.298 ***	−0.119	−0.164
Use of social-emotional support	−0.026	−0.040	−0.173	−0.069	−0.105
Positive interpretation and growth	0.282 **	−0.329 ****	−0.361 ***	−0.526 ****	−0.468 ****
Acceptance	0.081	−0.187 *	−0.043	−0.285 **	−0.202 *
Religious approach	0.001	0.112	0.103	0.044	0.092
Denial	−0.188 *	0.266 **	0.245 **	0.347 ****	0.335 ****
Expressing the emotions	−0.357 ****	−0.329 ****	0.309 **	0.309 **	0.354 ****
Mental deactivation	−0.286 **	0.362 ****	0.241 **	0.326 ****	0.353 ****
Behavioral deactivation	−0.452 ****	0.525 ****	0.501 ****	0.642 ****	0.640 ****
Substance abuse	−0.033	0.118	0.178 *	0.175	0.154
Sleeping	0.580 ****	−0.387 ****	−0.201 *	−0.314 ****	−0.357 ****
Speech	0.577 ****	−0.324 ****	−0.248 **	−0.406 ****	−0.380 ****
Mental	0.730 ****	−0.544 ****	−0.344 ****	−0.531 ****	−0.571 ****
Depression	0.699 ****	−0.662 ****	−0.373 ****	−0.588 ****	−0.652 ****
Distress	0.744 ****	−0.633 ****	−0.256 **	−0.482 ****	−0.549 ****
Vitality	0.758 ****	−0.653 ****	−0.372 ****	−0.497 ****	−0.593 ****
Sex	0.628 ****	−0.609 ****	−0.292 **	−0.405 ****	−0.516 ****
QoL	1	−0.751 ****	−0.429 ****	−0.641 ****	−0.725 ****

Significant correlation at the 0.05 level (two-tailed), *, *p*-value < 0.05; **, *p*-value < 0.01; ***, *p*-value < 0.001; ****, *p*-value < 0.0001.

## Data Availability

The data are available upon request from the corresponding author.
